# Safety and effectiveness of saving sphincter procedure in the treatment of chronic anal fissure in female patients

**DOI:** 10.1186/s12893-021-01346-5

**Published:** 2021-09-24

**Authors:** Beatrice D’Orazio, Girolamo Geraci, Sebastiano Bonventre, Dario Calì, Gaetano Di Vita

**Affiliations:** 1 General Surgery Unit, Department of Surgical, Oncological and Stomatological Sciences, University of Palermo, Via Liborio Giuffrè, 5, 90127, Palermo, Italy; 2 Postgraduate Medical School in General Surgery, University of Palermo, Palermo, Italy

**Keywords:** Proctology, Fissurectomy, Anoplasty, Anal fissure, Female patients

## Abstract

**Introduction:**

Lateral internal sphincterotomy (LIS) is still the approach of choice for the treatment of chronic anal fissure (CAF) regardless to the internal anal sphincter tone but it is burdened by high risk post-operative faecal incontinence (FI). In female patient there are some anatomical and functional differences of the sphinteric system which make them more at risk of FI and vaginal birth could cause sphinteric lesions affecting the anal continence function. The aim of our study is to evaluate the results of saving sphincter procedure as treatment for female patients affected by CAF.

**Methods:**

We studied 110 female patients affected by CAF undergone fissurectomy and anoplasty with V–Y cutaneous flap advancement associating pharmacological sphincterotomy in patients with hypertonic IAS. The follow up was at least for 2 years. The goals were patient’s complete healing, the evaluation of FI, recurrence rate and manometry parameters.

**Results:**

All wounds healed within 40 days after surgery. We recorded 8 cases of recurrences 6 healed with medical therapy and 2 with dilatation. We recorded 2 “de novo” temporary and low grade post-operative cases of FI. Post-operative value of MRP were unmodified in patient with normotonic IAS but significantly lower at 12 months follow up as compared with the pre-operative ones in patients with hypertonic IAS; after 24 months from surgery MRP values were within the normal range.

**Conclusion:**

The fissurectomy and anoplasty with V–Y cutaneous flap alone or in association with a pharmacological sphincterotomy in patients with hypertonic IAS may represent an effective approach for the treatment of CAF in female patients.

## Introduction

Chronic anal fissure (CAF) is a frequently occurring proctology lesion, which presents itself as a painful superficial tear in the cutaneous- mucosal transition zone of the anal canal. First line therapy is medical treatment with a dietary-higenyc and pharmacological approach. Nevertheless, in the event of CAFs, which are refractory to dietary and pharmacological therapy, lateral internal sphincterotomy (LIS) is still suitable as the treatment of choice independently from IAS tone. In fact, this latter surgical procedure is characterised by a low rate of post-operative complications and a high healing rate. The greatest disadvantage of this latter procedure is represented by the high rate of faecal incontinence occurrence (FI), which accounts for up to 30–40%. A meta-analysis from 2013, which evaluated long-term incidence of FI after LIS, showed an overall continence alteration risk of 14% [1]; however, on severity analysis, flatus incontinence and soilage/seepage were much commoner than frank incontinence to liquid or solid stool [2]. FI has a strong impact on the quality of life of patients and it can be more disabling than CAF itself; actually, patients tend to better bear a recurrence than FI [3]. From the guidelines for CAF treatment from different countries [4–9] we understand that the risk factors for FI occurrence are female gender, old age and the absence of an hypertonic IAS. In female patient there are some anatomical and functional [10] of the sphinteric system as compared to male patients, which make them more at risk of FI after proctological procedures; moreover, with vaginal birth sphinteric lesions may occur, which could affect the anal continence function. To reduce the risk of post-operative FI, the mostly used surgical procedure is fissurectomy alone or in association with pharmacological sphincterotomy and/or cutaneous or mucosal flap advancement.

The aim of our study is to evaluate the results of fissurectomy and anoplasty with V–Y cutaneous flap advancement in female patients affected by CAF.

## Materials and methods

This retrospective study involves 110 consecutive female patients, all affected by idiopathic and non-recurrent CAF who underwent surgical procedure, from January 2010 to January 2019. Exclusion criteria for the study were: the presence of multiple fissures, fistulas in ano, syphilis, inflammatory bowel disease, anal abscess, malignant disease and previous anorectal surgery. All patients were followed up for at least 2 years after the surgical procedure. The patients’ outcome data were retrieved from a prospectively monitored database.

We conduct this study in compliance with the principles of declaration of Helsinki, the protocol for this study has been submitted to the Ethical Committee of our institution, which did not consider necessary to approve it. Written informed consent was obtained from all the study participants.

Preoperative manometric evaluation was performed after a reasonable period of suspension of all medical therapy influencing IAS tone. The manometric evaluation was carried out by a manometric sensor using the station pull-through as described in our previous work [11]. A manometric evaluation has been undertaken at 12 and 24 months after surgery.

Data collected on healthy subjects by our anorectal pathophysiological laboratory showed that normal values of maximum resting pressure (MRP) and maximum squeeze pressure (MSP) were respectively 68.1 ± 12.3 mmHg and 112 ± 36.2 mmHg [11]. The normal range of MRP, according to Jones et al. [12], were 45–85 mmHg; so that CAF without hypertonic IAS were defined as those with MRP values < 85 mmHg.

All patients underwent fissurectomy and anoplasty with V–Y skin flap advancement lying in a gynecological position under spinal or general anesthesia.

The sentinel skin tags and hypertrophied anal papilla located at the dentate line were excised, if present; the tissue at the base of the fissure was curetted until clean IAS muscle fibers were reached. Technical details concerning the surgical procedure have already been widely explained in a previous work from our group [13].

The patients with hypertonic IAS were treated with intraoperative local injection of 30 U.I. of botulinum toxin A (Botox, Allergan Westport, Ireland) [14] or with local administration of post-operative nifedipine and lidocaine for 15 days after surgery (Antrolin ®) [15]. Before surgery, all patients received a small volume of phosphate-saline enema. Metronidazole was administered intravenously in a dose of 500 mg 1 h before surgery, subsequently, it was administered per os at the dosage of 250 mg for 7 days, three times daily. During the first two weeks after the surgery, patients took variable doses of psyllium fibers. A laxative preparation (sennosides) was given orally to subjects who had not yet passed stools 3 days after surgery. Immediately after surgery, all patients received 100 mg of diclofenac intramuscularly for analgesia and were instructed to take only 100 mg of nimesulide tablets as requires.

A complete healing was defined as a complete epithelialization of the advancement skin flap. Recurrent CAFs were defined as those who occurred after the complete healing of the previous wound. Both duration and intensity of post-defecatory pain have been evaluated; the intensity was evaluated with a visual analogue scale (VAS).

FI was assessed preoperatively and 6, 12 and 24 months after surgery according to Pescatori’s grading system [16]: A incontinence for flatus and mucus; B for liquid stool; C for solid stool; 1 for occasional; 2 for weekly and 3 for daily. Patients were discharged within 24 h after surgery, afterwards they were examined until they were completely healed with a first appointment after 3 days from surgery, a second on post-operative day 10 and on a regular basis each 10 days for the first two months after surgery. They were also followed up until 24 months following the surgical procedure. Independently of the scheduled appointments, patients have been seen on request.

### Statistical analysis

Data were analyzed by standard statistical methods and the results were expressed as means ± standard deviation. Differences between continuous data were compared using Student t test for paired and unpaired samples, whereas differences between percentages were analyzed using Fisher test. Probability values of < 0.05 were considered significant.

## Results

Demographic, clinical characteristics of CAF of the patients object to study are reported in Table 1. We observed an anterior location of the CAF in the 34.5% of patients, while we recorded an hypertonic IAS in the 65.5% of them (Table 2). Among the 72 patients with hypertonic IAS, we treated 40 of them with the injection of Botulinum Toxin A, while 32 of them with topical administration of nifedipine and lidocaine ointment. At 40 days after the surgical procedure we achieved a complete wound healing a resolution of clinical symptoms. The duration and intensity of post-defecatory pain have significantly reduced as compared with the pre-operative values since the first defecation (Table 3).

**Table 1:** Clinical and demographic characteristics of CAF

	No.	%
Gender		
Female	110	100
Age (mean ± standard deviation)	36.5 ± 15.8	
Hypertrophied anal papilla	88	80.0
Skin tags	82	74.6
Symptoms		
Pain	110	100
Bleeding	88	80.0
Pruritus	66	60.0
Duration of symptoms		
Months (mean ± standard deviation)	12.1 ± 7.3

**Table 2:** Location and IAS tone in the patients object to study

Location	Normotonic IAS		Hypertonic IAS		Total	
	N°	%	N°	%	N°	%
Anterior	21	55.3	17	44.7	38	34.5
Posterior	17	23.6	55	76.4	72	65.5
Total	38	34.5	72	65.5	110	100

**Table 3:** Intensity of pain evaluated by VAS and duration of pain expressed in minutes after defecation

	Intensity of pain	Duration of pain
Preoperative	8.5 (1.3)	165 (40.3)
1st defecation	4.2 (1.5)*	100 (35.1)*
3rd defecation	4.1 (1.1)	60.5 (20.8)
5th defecation	3.0 (0.6)	30.2 (12.3)
7th defecation	2.2 (0.7)	10.4 (5.1)
9th defecation	0.8 (0.3)	5.1 (2.8)
10th defecation	0.2 (0.4)	2.8 (0.7)

Values are expressed as mean and standard deviation. Student’s *t* test with Welch correction was used to compare the difference between each point. Significance. 1st defecation vs preoperative *p < 0.01

### Manometry findings

At 12 and 24 months after surgery MRP and MSP values were substantially unmodified in the patients with normotonic IAS (Table 4). In those patients with hypertonic IAS MSP values were immodified as compared to the values at 12 and 24 months after surgery; whereas MRP ones were significantly lower at 12 months follow-up as compared to the pre-operative ones but still significantly higher than those measured in healthy controls. At 24 months follow up after surgery MRP values were within normal range (Table 5).

**Table 4:** MRP and MSP values as mean ± standard deviation in patients with normotonic CAF before and after the surgical procedure

	MRP (mmHg)	MSP (mmHg)	p
Healthy subjects	68.1±12.3	112±36.2	n.s.
Pre-operative	59.3±15.1	107±26.4	
12 months after surgery	63.5±18.6	110±22.3	
24 months after surgery	61.3±12.1	103±17.1

**Table 5:** MRP and MSP values as mean ± standard deviation in patients with hypertonic CAF vs healthy subjects before and after the surgical procedure

	MRP (mmHg)	p	MSP (mmHg)	p
Healthy subjects	69.8 ± 11.8		112 ± 36.2	
Patients with CAF				
Pre-operative	95.3 ± 8.5	0.0001	109.2 ± 46.5	n.s.
12 months after surgery	77.3 ± 15.2	0.0018	115.1 ± 28.9	
24 months after surgery	70.2 ± 10.5	n.s.	107.9 ± 31.9	

### Faecal incontinence

We recorded 15 cases of pre-operative FI (13.63%), in 7 of them CAF was located at the anterior commissure (18.42%), in 8 of them at the posterior one (11.11%). FI cases and rate recorded pre-operatively related to childbirths are reported in Tables 6 and 7 and those related to the location in Table 8.

**Table 6:** Faecal incontinence assessed preoperatively related to childbirths

	Childbirth		Faecal incontinence	
	N°	%	N°	%
Nulliparous	38	34.5	3	7.8
Multiparous	72	65.6	12	16.6

**Table 7:** Faecal incontinence assessed preoperatively related to the type of delivery

	Childbirth		Faecal incontinence	
	N°	%	N°	%
C-section	27	24.5	3	11.1
Vaginal birth	45	40.5	9	20

**Table 8:** Faecal incontinence assessed preoperatively related to CAF’s location

Location of the CAF	Patients		Faecal incontinence	
	N°	%	N°	%
Anterior	38	34.5	7	18.42
Posterior	72	65.6	8	11.1

According to Pescatori’s grading system [17] 10 were classified as A1, 4 as A2 and 1 as A3. We observed only 2 “de novo” case of post-operative FI both classified as A1 and temporaries. We recorded a temporary worsening of A1 pre-operative grading in 2 cases: in the first one it occurred in patients treated with Botulinum Toxin A injection and the CAF was located at the posterior commissure; in the other one the IAS tone was normal and the CAF located at the anterior commissure.

### Recurrence

At 24 months after the surgical procedure follow-up we recorded 8 recurrences (7.3%). In 6 of them MRP values were normal and they were successfully treated with conservative therapy (pharmacological and hygienic-dietary approach). In 2 cases there was a mild hypertonic IAS, these latter have been treated with dilatation with complete healing.

### Complications and follow-up

We did not observe any anal stenosis nor keyhole deformity. At the same time, we did not record any necrosis of the transposed flap. The post-operative complications we were able to observe were of slight entity and they didn’t require, in any case, further surgical procedures; in particular we observed 1 case of infection located at the donor site as well as 8 case of partial break down.

## Discussion

The results of this study show that fissurectomy and anoplasty associated with a pharmacological therapy in case of hypertonic IAS is feasible and effective; this procedure allows a perfect post-operatory faecal continence and a low rate of recurrence.

LIS is the treatment of choice for CAFs, but in female patient both of childbearing age or elderly it is not appropriate to undertake this procedure, nor in the traditional nor in the new proposed variant [17–23]; due to the high risk of immediate or long term post-operative FI, caused by some anatomical, clinical and functional features typical of these patients as compared to male ones, which expose female patients to a higher risk of FI [24].

Murad-Regadas et al. [25], through a 3-D anal endosonography have demonstrated an asymmetrical configuration of anal canal and a difference between the two genders. In women patients both external anal sphincter (EAS) and IAS are shorter at the anterior level and they are characterized by a longer gap, which may justify the higher incidence of pelvic disfunction. It is still not known whether some women have slightly different pressure distribution in the distal anal canal predisposing to a higher risk than man for the development of anterior CAF [26]. Ellis [27] reported that patients affected by anterior CAF are frequently affected also by rectocele at physical examination with a typical manometric profile (high pressure zone shortened with low to normal resting pressure).

We shall consider that female patients of childbearing age could give birth by vaginal delivery during which the lesions of the anal sphincteric system may occur. Sultan et al. [28], report in their study that an isolated disfunction of the IAS might occur after vaginal delivery in the 13% as well as a disfunction of EAS in the 59% of cases, while a disfunction of both sphincters arrives in the 10% of cases. Richter et al. [29] observed a sphincteric disfunction after vaginal delivery in the 35% of cases. Another risk factor recently took into account for the occurrence of CAF of the anterior commissure is the usage of water-jet stream in a bidet-toilet, which according to Garg [30] strongly increase the incidence of this condition. Moreover, we conducted a review of the literature showing an incidence of CAF of the anterior commissure as high as 35.4% [31], this data owes its justification to the high number of multiparous women who gave birth by vaginal delivery along with the routine habit of using water-jet stream in a bidet toilet [30].

As for the differences in manometric parameters in various research, different authors [32–34] report that MSP values are significantly lower in female patients, as they represent the strength of the EAS, which is made by striated muscle. As a matter of fact, men have a greater striated muscle due to the well-known effect of testosterone of muscle growth [34]. MRP values, on the contrary, are similar in both sexes [32–35].

It appears logical to associate a chemical sphincterotomy for those patients who have hypertonic IAS, aiming to reduce the IAS tone and improve the IAS blood perfusion. We include various drugs fit for purpose, among these we considered Botulinum Toxin A and nifedipine. The first one has been injected during the surgery, therefore ensuring a high level of compliance; nevertheless, it is burdened by an augmented risk of FI, as we see in this study in one case, moreover, its employment in the treatment of CAF it is considered off label in Italy. On the other hand, nifedipine is employed by topical administration 15 days after surgery, therefore we experienced lower compliance as well as less side effects including FI. Both Nifedipine and Botulinum toxin A induce relaxation of the IAS tone with different mechanism. Botulinum toxin A reduces the release of noradrenaline at the neuromuscular junction of the peripheral nerves [36], inducing a constant relaxation of the smooth muscles without interfering with the volunteer control of the continence [37]. The latter effect develops in few hours and the contraction start again within 3 or 4 months after the emergence of a new neuromuscular junction [37]. The nifedipine might be due also to its anti-inflammatory action not only to its induced reduction of the IAS tone [20, 38–42], carried through the inhibition of calcium flow into the sarcoplasm of IAS. Experimental studies indicate that nifedipine has a modulating effect on microcirculation [43], as well as a local anti-inflammatory effect [44]. Moreover, nifedipine might contribute to the healing process of CAF due to its additional free radical scavenging properties [45]. In our study we didn’t observe any adverse effects related to the employ of nifedipine such as headache or perianal dermatitis, patients’ compliance has been excellent. Available research focusing on LIS outcomes in female patients are few. Regadas et al. [46] and Fernandes et al. [47] analyzed respectively a series on 31 and 36 female patients affected by CAF hypertonic IAS, without FI or previous proctological procedure, who underwent LIS extended to the apex of the fissure; they observed, in the 52–53% of cases. The authors highlighted, with a post-operative evaluation endoanal ultrasound (EAUS), that if LIS extension was greater than 20–25% of the IAS length (corresponding to less than 1 cm), the incidence of FI was significantly higher. It appears difficult, if not impossible, to divide exactly a quarter of the IAS length, as there is not any reference landmark neither with EAUS.

In their recent research Brillantino et al. [48] performed a sphincterotomy limited to the 20% of the IAS length as treatment for female patient affected by CAF with hypertonic IAS and without any clinical sign of FI, with the help of a new device introduced by themselves. This latter procedure requires a pre-operative evaluation with an EAUS and the researchers achieved very good results with a rate of healing of the 93.3% and a rate of recurrence of 6.2% without alteration of the anal continence. Nevertheless, this proctological surgery appears complex and requires equipment, which are not available in every hospital.

Even if a LIS limited to the 20% of the IAS length allows to achieve good results both in terms of healing and FI rate, in female patients affected by CAF with hypertonic IAS without signs of FI or previous proctological procedure, this procedure appears difficult and requires further technical development. So that, up to date sphincter saving surgical procedure represent the treatment of choice for female patients affected by CAF regardless of the IAS tone, age, previous proctological procedure or presence of FI [49–53]. In fact, as for female patients a sphincter saving procedure appears mandatory given the higher risk of FI; these latter aim to perform a fissurectomy along with a wound debridement, removing the bradytrophic scar tissue, producing a fresh wound edges and finally creating an acute fissure. The subsequent employ of a flap to cover up for the naked area is designed to relocate on this area healthy and fresh blood supplied tissue, perfused by other arterial district [14].

## Conclusion

The saving sphincter procedure we undertook, fissurectomy with V–Y advancement flap associated with chemical-pharmacological sphincterotomy for IAS tone reduction, in case of hypertonic IAS may represent an effective approach for the treatment of CAF in female patients. Only in case of failure of these latter LIS could be taken into account as an alternative approach.

## Data Availability

The datasets generated during and analysed during the current study are not publicly available because we did not consider it necessary to publish them in a public repository but are available from the corresponding author on reasonable request.
